# Scintigraphic Methods to Evaluate Alterations of Gastric and Esophageal Functions in Female Obesity

**DOI:** 10.4274/Mirt.14633

**Published:** 2014-02-05

**Authors:** Özgür Ömür, Mehmet Erdoğan, Hayal Özkılıç, Candeğer Yılmaz

**Affiliations:** 1 Ege University Faculty of Medicine, Department of Nuclear Medicine, İzmir, Turkey; 2 Ege University Faculty of Medicine, Department of Endocrinology and Metabolism, İzmir, Turkey

**Keywords:** obesity, esophageal motility disorders, gastroesophageal reflux

## Abstract

**Objective**: Altered gastrointestinal function has frequently been observed in obese patients. The aim of this study was to investigate the frequency of gastro-esophageal reflux (GER) and to determine the alterations of gastric emptying and esophageal transit by scintigraphic methods in obese patients.

**Methods**: Scintigraphic studies of 50 obese female non-diabetic patients who had not received any treatment for weight control were retrospectively reviewed. Mean Body Mass Index (BMI) was 34.96±3.04 kg/m^2^ (range:32-39 kg/m^2^). All subjects were submitted to scintigraphic evaluation of esophageal transit, gastro-esophageal reflux, gastric emptying and presence of Helicobacter pylori infection. The data of obese patients were compared with those of sex-age matched 30 non-obese cases who were selected from our clinical archive.

**Results**: In obese group, seventeen (34%) patients were found to be GER positive scintigraphically; mean gastric emptying time (t½) was 59.18±30.8 min and the mean esophageal transit time was 8.9±7.2 s. Frequency of positive GER scintigraphy and the mean value of esophageal transit time were significantly higher in obese patients than non-obese control subjects. Gastric emptying time and esophageal transit time values were significantly longer in GER positive obese patients than GER negative ones. There was no statistically significant difference in the frequency of positive C14 urea breath test between obese and non-obese subjects and there were also no statistically significant correlations between BMI, GER, esophageal transit time and gastric emptying time.

**Conclusion**: In our study, 42 of the 50 obese patients had esophago-gastric motility alterations. The significance of these alterations in obesity is not fully understood, but it is believed that these changes could be because of potential contributing factors in the development or maintenance of obesity or changes in eating habits.

**Conflict of interest:**None declared.

## Abstract (tr)

**Amaç**: Obezitede gastrointestinal fonksiyon değişiklikleri sıklıkla meydana gelmektedir. Bu çalışmada obez olgularda gastroözefageal reflü (GÖR) sıklığını araştırmak, mide boşalım ve özefagus transit zamanlarındaki değişiklikleri sintigrafik yöntemlerle değerlendirmek amaçlandı.

**Yöntem**: Kilo kontrolüne yönelik tedavi almayan, diyabetik olmayan 50 kadın obez olguya ait veriler retrospektif olarak incelendi. Ortalama Vücut Kitle İndeksi (BMI) 34.96±3.04 kg/m^2^ idi (değer aralığı: 32-39 kg/m^2^). Tüm olgularda özefagus transit ve mide boşalım zamanları, gastroözefageal reflü varlığı sintigrafik yöntemlerle değerlendirildi. Elde edilen veriler klinik arşivimizde bulunan yaşları uyumlu obez olmayan 30 kadın olguya ait sonuçlar ile karşılaştırıldı.

**Bulgular**: Obez grupta 17 olguda (34%) sintigrafik olarak gastroözefageal reflü saptandı. Ortalama mide boşalım yarı zamanı (t½) 59,18±30,8 dk, ortalama özefagus transit zamanı ise 8,9±7,2 saniye idi. GÖR sıklığı ve ortalama özefagus transit zamanı obez grupta kontrol grubuna gore anlamlı yüksek bulundu. Mide boşalım ve özefagus transit zamanları GÖR saptanan obez olgularda, GÖR saptanmayanlardan anlamlı yüksekti. Obez ve obez olmayan olgular arasında C14 üre nefes testi pozitifliği açısından anlamlı fark bulunmadı. Vücut kitle indeksi ile GÖR, özefagus transit ve mide boşalım zamanları arasında anlamlı korelasyon saptanmadı.

## INTRODUCTION

Obesity is a medical condition in which the body mass index (BMI) of a person exceeds 30 kg/m^2^. It has become the most common chronic health problem in many countries. In the United States alone, obesity is currently the second leading cause of preventable death, with more than 50% of adults estimated as being overweight or obese and 5% as being morbidly obese (1,2,3). Obese individuals have increased incidence of medical problems including coronary artery disease, hypertension, peripheral vascular disease, pulmonary insufficiency, sleep apnea, diabetes, osteoarthritis, and gastro-esophageal reflux disease (GERD), together with psychosocial disabilities.

GERD is the most common pathologic condition of the foregut in the Western World and accounts for about 75% of all esophageal diseases (4,5). Obesity and GERD show a prevalence of 30% and 15-20%, respectively, in adults in Western countries (6,7). Obesity is a predisposing factor to GERD, but in obese patients, esophageal function still remains poorly studied (8). The evaluation of esophageal physiology in laboratory has been increasingly used to understand and manage symptoms such as heartburn, regurgitation, atypical reflux symptoms, chest pain, and dysphagia. Among several diagnostic techniques, esophageal manometry and 24-h pH monitoring are still employed in the clinical practice (9,10,11). Altered gastrointestinal functions, esophageal motility disorders and a higher incidence in the Helicobacter pylori infection have been also reported in obese subjects (12,13,14,15,16,17,18,19). In this study, we aimed to evaluate the presence of GERD, Helicobacter pylori infection and gastric-esophageal motility alterations in obese patients by nuclear medicine methods.

## MATERIALS AND METHODS

**Patients**

Scintigraphic studies of 50 non-diabetic obese (BMI≥30) female patients who had not received any treatment for weight control or gastro-esophageal symptoms were retrospectively reviewed. The mean age was 49.6±8.1 years and the mean BMI was found as 34.96±3.04 kg/m^2^ (range: 32-39 kg/m^2^). Patients neither have any other systemic disease nor have any medications that may affect gastro-esophageal functions. All subjects were submitted to scintigraphic evaluation of esophageal transit, gastroesophageal reflux and gastric emptying. C-14 urea breath test was also performed to all of the patients to assess the presence of helicobacter pylori infection. Informed consents were obtained from all patients. The data of obese patients were compared with those of sex-age matched 30 non-obese cases who were selected from our clinical archive.

**Scintigraphic Studies**

**Scintigraphic Evaluation of Esophageal Transit**

The subjects were studied while sitting in front of the collimator of the gamma camera, facing forward, and head at midline during the scintigraphic examination. All patients were given 15 ml water containing 0.5 mCi (18.5 MBq) Tc-99m labeled diethylene triamine pentaacetate (DTPA). The patients were instructed to hold the mouthful of the prepared water in their mouth for a few seconds and then swallow it when the examiner gave a sign. Dynamic acquisitions in a 64x64 matrix were performed with large-field-of-view gamma camera (Toshiba GCA-602A) equipped with low energy all-purpose parallel hole collimator (LEAP). During the study, 20 transit images were recorded at 0.5 s intervals. Regions of interests (ROIs) were outlined on the esophagus and then the time-activity curves (TAC) were created. From this TAC, esophageal transit time was calculated using a software programme. The observation of the peak activity on the TAC within 10 s was accepted as normal esophageal clearance.

**Scintigraphic Evaluation of Gastro-esophageal Reflux**

Gastro-esophageal reflux scintigraphy was performed after esophageal transit study without giving the patient any other radioactivity. Following the esophageal transit study, the patients were given additional water to washout oropharyngeal and esophageal residual activity. After this procedure, patients were re-positioned supine under the gamma camera (Toshiba GCA-602A) equipped with a LEAP collimator. Dynamic imaging was performed in 64x64 matrix with 60-s frames for 30 min. TACs were derived from ROIs drawn over the esophagus and stomach. TACs were analyzed and additionally all images were visually evaluated. Detection of the activity in the esophagus on the images visually or detection of one or more peaks on TACs at any time during the scintigraphic evaluation was considered as gastro-esophageal reflux. Peaks which displayed a two-fold or more increment in the esophageal counts over the baseline were accepted as reflux episode.

**Scintigraphic Evaluation of Gastric Emptying**

Scintigraphic assessment of solid phase gastric emptying was performed in fasting state for at least 4 h. The test meal composed of an egg sandwich (bread approximately 200 g) labeled with 0.5 mCi (18.5 MBq) Tc-99m DTPA. The patients were asked to eat the sandwich within 5 min. Immediately after ingestion of the meal, patients were positioned supine under a large field-of-view dual headed gamma camera (Sophy DST) equipped with LEAP collimator. Attenuation and decay corrections were made by software program. Images were recorded in a 64x64 matrix in 30-s frames for 60 min. After 60th min, planar images were obtained with an interval of 15 min until 2th, consecutively. TACs were created from an irregular ROI drawn over the stomach and geometric mean of the anterior and posterior gastric counts was used. Gastric emptying was expressed as the half time of emptying (t1Ğ2) and determined as the time it takes to reach half of the peak counts calculated from TAC.

**C14 Urea Breath Test**

Medications such as antibiotics and bismuth compounds were withdrawn 30 days before the test while withdrawal period for sucralfate and proton pump inhibitors were two weeks. C14 urea in a capsule form containing 1 mg urea labeled with 37 kBq (1 μCi) C14 was used for the test after at least 6 h starvation. Patients swallowed this capsule with 20 ml of water. At 10 min post-dose, patients were asked to take a deep breath, hold it for approximately 5-10 s and then exhale through a straw into a Mylar balloon. Balloons with breath samples were calculated in the scintillation counter. Reference values are as follows: <50 dpm: Negative, 50-199 dpm: Indeterminate and ≥200 dpm: Positive for H. pylori. If a value of 50-200 dpm was obtained, the samples were recounted in 1-2 h or the next day.

**Statistical Analysis**

The mean values and standard error of the means of all measured quantities were calculated. Clinical and scintigraphic parameters of subgroups of the patients such as gastric emptying and esophageal transit half times, BMI and age were compared using 2-tailed Student t test for unpaired samples. Correlation between GER and C14 urea breath test positivity, gastric emptying-esophageal transit half time or BMI was done with Spearman’s correlation test. Differences between some numerical parameters of the obese and non-obese control subjects were evaluated using Fischer’s exact test. P<0.05 is considered as statistically significant.

## RESULTS

In 50 obese cases, 42 patients had one or more positive scintigraphic findings associated with gastrointestinal system. Seventeen of 50 obese patients were found to be GER positive scintigraphically and 29 patients had positive C14 urea breath test. In non-obese control group, 4 cases were positive for GER and 14 cases were positive for C14 urea breath test. The mean gastric emptying time (t½) and the mean esophageal transit time was 59.18±30.8 min and 8.9±7.2 s in obese subjects, respectively, while they were 50.3±29.2 min and 6.9±3.4 s for non-obese case group, respectively. Frequency of GER positivity and the mean esophageal transit time were significantly higher in obese patients than non-obese cases (p=0.02 and 0.04 respectively). The summary of the clinical and scintigraphic parameters of the obese patients and non-obese subjects are presented in Table 1.

According to the comparison made between GER positive and GER negative patients (Table 2), the mean gastric emptying time was found as 64.9±29.2 and 53.3±30.2 min, respectively.

The comparison of gastric emptying time between GER positive and negative patients was statistically significant (p=0.03). The mean esophageal transit time was also significantly longer in GER positive patients (11.2±7.3 s) than GER negative patients (8.2±7.2 s) (p=0.039). Positive correlations were determined between GER positivity and t½ values of gastric emptying and esophageal transit, which were significantly longer in GER positive obese patients (p=0.036).

In 19 of the 50 obese patients, gastric emptying time (mean: 41±3.6) was shorter than normal reference values (<45 min). In these patients the mean esophageal transit time was 7.89±7.13 s and the rate of GER positivity (%15) was lower than those with a normal reference values and prolonged gastric emptying time. The frequency of positive C14 urea breath test was significantly higher in these patients than that of the prolonged gastric emptying (p=0.02) (Table 3). Gastric emptying time was longer than normal reference values in 5 obese patients. In these cases, mean esophageal transit time and frequency of GER positivity were significantly higher; while C14 urea breath test positivity was lower than the other groups (Table 3).

In 13 of 50 obese patients, esophageal transit time was longer than 10 s and 7 of these patients were found to be GER positive. There was no statistically significant difference between gastric emptying time and C14 urea breath test positivity in patients with normal and prolonged esophageal transit time (Table 4).

No statistically significant correlation was found considering the relationship between BMI and other variables such as GER positivity, esophageal transit time, and gastric emptying time.

## DISCUSSION

Gastrointestinal (GI) system motility plays a critical role in consumption of foods, digestion and absorption of nutrients. GI motility not only regulates the absorption of nutrients, but also participates in the control of appetite (12). While the significance of altered GI functions in obesity is not fully understood, altered GI motility has been frequently observed in obese patients and suggested to be important in the development of obesity and eating attitude (12). Among several co-morbidities associated with obesity and GERD has been reported to be prevalent also (13,14,15,16). Esophageal motility disorders and altered gastric functions may be accompanied by GERD in obese patients (16,17,18,19). Nearly all epidemiologic studies have found an association between increasing BMI and symptoms of GERD. Changes in gastro esophageal anatomy and physiology caused by obesity may explain the association (13,14,15,16,17,18,19). These include an increased prevalence of esophageal motor disorders, diminished lower esophageal sphincter (LES) pressure, the development of a hiatal hernia and increased intragastric pressure (20). Additionally, metabolic, hormonal or behavioral factors may be involved in the etiology of obesity.

Gender differences in obesity and its effect on GI functions were also evaluated in the literature. Delayed gastric emptying which was assessed using non-invasive 13C-octanoic acid breath test (21), slower gastric emptying in patients taking oral sex hormone replacement (22), higher incidence of GERD (23) and increased risk of esophageal disorders such as adenocarcinoma, Barrett esophagus and GERD predominantly abdominal or intra-abdominal adiposity (24) have been reported in female obesity.

Although there were several studies about GERD, esophageal dysmotility and gastric functions in obese patients, to the best of our knowledge there was no study evaluating combined gastric-esophageal functions and GERD by scintigraphic methods in obese individuals. In this study, the presence of GERD, alterations of esophageal and gastric functions were investigated and the inter-relationship between all of these parameters with obesity was examined.

Our results revealed that the presence of GER in obese patient group (34%) was significantly higher than that of non-obese cases (13%). The mean gastric emptying time was as expected in significantly higher but normal reference ranges in GER positive obese patients when compared to GER negative obese patients (64.9±29.2 min vs. 53.3±30.2 min, respectively). The mean esophageal transit time was also significantly longer in GER positive obese patients (11.2±7.3 s) than GER negative obese patients (8.2±7.2 s) and non-obese cases (6.9±3.4 s). It was shown by different studies that gastric emptying and esophageal transit were significantly delayed in patients with GERD (25,26). In this study, the mean gastric emptying time was not prolonged in whole study group and in the subgroup of GER positive patients. However, number of patients with delayed gastric emptying and the mean gastric emptying time were significantly higher in GER positive patients (Table 2). It may be speculated that the motility abnormalities that cause GERD can also cause delayed gastric emptying by the similar mechanism or contrarily delayed gastric emptying may contribute to the development of GERD. Although there were various studies about esophageal motility abnormalities after gastric banding surgery in obese patients, the evaluation of esophageal functions in non-operated obese cases especially using radionuclide methodologies still remains insufficient (6,8).

In agreement with our results, Mercer CD et al. have reported that the esophageal transit time as measured by radionuclide methods was significantly prolonged in obese group as compared with normal population (27). But a factor that may affect esophageal transit, GER, has not been evaluated in this study. Additionally, increased transient lower esophageal sphincter (LES) relaxation, stronger peristalsis, increased acid exposure regardless of presence of GERD have been reported in obese cases (11,28). In obese patient group of our study, the mean esophageal transit time (8.9±7.2 s) was not longer than the normal reference (<10 s), but significantly longer than that of non-obese subjects. Thirteen of 50 obese patients had delayed esophageal transit time. Nearly half of these patients had GER and it’s remarkable that the mean esophageal transit time in obese patients with GER was found significantly longer than GER negative cases (Tables 3 and 4). Gastric emptying time was also significantly longer in patients with delayed esophageal transit than the others. For this reason, contribution of GER on esophageal transit and gastric functions was considered in obese patients. Several studies proved that the development of GERD was usually associated with a decreased LES pressure, increased transient LES relaxations and decreased esophageal clearance capacity (8,11,12).

Accelerated gastric emptying was found in 19 of 50 obese cases (38%). Most of the studies that investigate gastric motility in obese patients have also reported accelerated gastric emptying of solids (12,19,29,30). In the current study, there was no significant difference in mean gastric emptying time in obese and non-obese subjects. Similarly, no significant association was determined between esophago-gastric parameters or presence of GER and BMI. Our patient group to be uniform in terms of age and BMI may lead to this situation.

In this study group, 29 of the 50 obese patients had positive C14 urea breath test as the indicator of Helicobacter pylori infection. There was no significant association between C14 urea breath test positivity and esophageal transit or presence of GER. However, the number of patients with positive C14 urea breath test was significantly higher in subgroup of patients with shorter gastric emptying half time than longer ones. Epidemiological studies suggested that the frequency of helicobacter pylori infection in asymptomatic population ranged from 10% to 60% (31). Metabolic and endocrinological syndromes or obesity that might be accompanied with Helicobacter pylori infection have also been reported (15,32,33). Al-Akawaa AM noted that prevalence of Helicobacter pylori infection in morbidly obese Saudi patients was about 85% (34). Although, C14 urea breathe test is the screening test for evaluating Helicobacter pylori infection, frequency of this infection (58%) was not so different in our obese cases than reported in normal population range (10-60%) and our non-obese control subjects (46%). These results may suggest that the lack of morbidly obese patients (BMI>40 kg/m^2^) in our study group could be responsible for this condition.

The major limitation of the study was gastric emptying time evaluations were based on the calculation of department normals. The lack of standardization of the method of analysis may cause some problems in interpreting results. Reported values of gastric emptying are influenced by the duration of testing and the method of analysis (35).

Taken together all these results suggest that esophageal and gastric motility alterations play an important role on pathophysiology of obesity. However, each patient may have different motility alterations and, therefore case based analysis is necessary for modification of the treatment strategy. As a conclusion, scintigraphic studies can be used for the evaluation of GI motility alterations in obese subjects as they are non-invasive, easily applicable methods with a very low radiation exposure.

## Figures and Tables

**Table 1:**
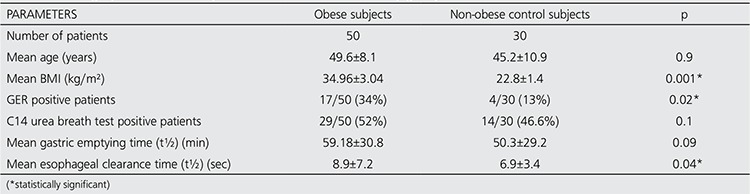
Scintigraphic and clinical parameters of the whole study group

**Table 2:**
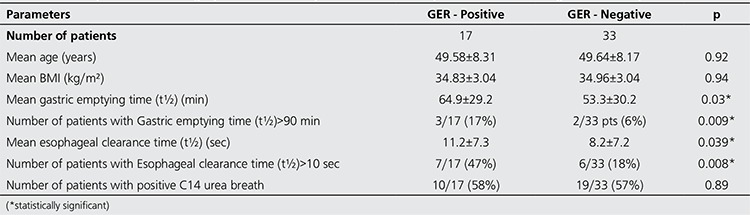
A comparison of scintigraphic and clinical parameters of GER positive and GER negative obese patients

**Table 3:**
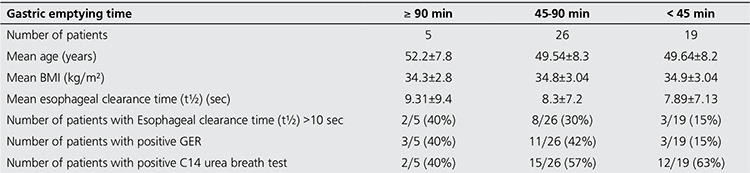
Characteristics of patient groups with gastric emptying time shorter and longer than normal reference values (45-90 min)

**Table 4:**
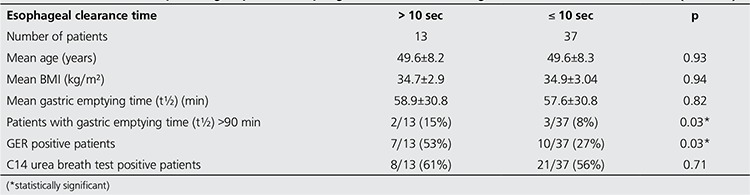
Characteristics of patient groups with esophageal clearance time longer than normal reference value (> 10 sec)
